# Resolving tissue and cellular functions of parasitic nematodes

**DOI:** 10.1016/j.pt.2025.09.012

**Published:** 2025-10-25

**Authors:** Paul M. Airs, María A. Duque-Correa

**Affiliations:** 1 Cambridge Stem Cell Institute, University of Cambridge, Cambridge, CB2 0AW, UK

## Abstract

Parasitic nematodes have a significant impact on global health as pathogens of humans and animals. Yet, our understanding of parasite physiology and the function of organs critical for intra-host survival is poor. Most knowledge is derived from *Caenorhabditis elegans*, a free-living nematode that has limited translatability to parasitic species. Here, we discuss opportunities to fill knowledge gaps in fundamental parasite biology through the study of parasite body plans, tissues, and cells in their biological context, using modern imaging, omics, metabolic models, and *in vitro* culture systems. Resolving the functions of parasite cells and tissues throughout development and inside their hosts is key to discovering new tools to tackle them.

## What is this organ for? The importance of functionally characterising parasitic nematode tissues

Parasitic nematodes are highly successful pathogens that significantly impact human and animal health as well as livestock production [1–4]. Paradoxically, there is much we still do not understand about the physiology and the interactions of these parasites with their hosts. Indeed, there is an urgent need to investigate aspects of parasite biology that are specific to each species and that enable the successful infection of their definitive hosts, to develop targeted and effective anthelmintics and vaccines as well as more accurate diagnostic tools.

Parasitic nematodes of mammals exploit a diverse range of life history strategies to invade and chronically infect their respective hosts. They sense and traverse body barriers (i.e., skin, lung, or gut), and some migrate through multiple tissues as they develop, before settling in a specific organ of their host – such as the intestine, abomasum, lung, or lymphatics – where they persist by modulating the host’s immune response [5–7]. Distinct life-cycle stages displaying particular morphological adaptations interact with specific intra-host environments. However, it remains unclear how the host environments have shaped the evolution of these adaptations and how they contribute to the parasite’s ability to survive in particular niches. These host–parasite interactions likely initiate signalling cascades that: (i) drive developmental transitions in the parasites, and (ii) trigger biological processes required for the colonisation of tissues within their definitive host. Thus, understanding how specific parasite organs develop across the life cycle and what function they play in the physiology of parasitic nematodes may provide important insight into key parasite traits.

Most of our knowledge on parasite tissues, cells, and mediators of host–parasite interactions is derived from whole worms isolated from their hosts. This approach is problematic because it can miss critical tissue-level aspects of the host–parasite interplay driving infections, such as cellular interactions and localised immune responses that influence parasite gene expression and mediator production. Further, while there is a growing body of literature demonstrating the importance of molecular exchanges between parasitic nematodes and their hosts, such as excretory–secretory (ES) products (see Glossary) [5,7], there is little understanding of how and where these molecules are produced and exchanged *in situ*. There is, therefore, a need for studies on the function of organs and cells of parasitic nematodes in the context of their host niches.

Work towards functionally characterising tissues of parasitic nematodes as they exist *in situ* and at the cellular and molecular level can now be achieved through the integration of novel technologies such as single-cell/single-nuclei and spatial transcriptomics, proteomics and metabolomics, organoids and other *in vitro* co-culture systems, and advanced microscopy [8–10]. The interpretation of data resulting from these techniques to infer tissue function could be greatly improved with accurate anatomical knowledge of the parasites. This knowledge is, however, outdated or missing, yet it is essential for localising key genes, proteins, and metabolites involved in parasitic processes such as attachment, migration, and immune evasion. Moreover, a better comprehension of parasitic nematode anatomy can reveal previously unrecognised structures and cell types that may be critical for a parasitic lifestyle. This opinion article highlights existing knowledge gaps in the functional anatomy of parasitic nematodes of humans and other mammals as barriers to a deeper understanding of these pathogens. We discuss whether structural conservation equates to functional conservation at the tissue level when parasites exist in specific host niches, and whether parallels can be effectively drawn between species, especially in reference to their free-living counterparts. Further, we propose potential avenues to close these knowledge gaps through the study of parasitic nematodes as they exist in space and developmental time in their host, identifying the limitations and challenges of different approaches. Finally, we urge the revision and update of seminal anatomical data and inclusion of morphometric studies of parasitic nematodes during development, exploiting modern imaging technologies (see later), as a stepping stone for the functional characterisation of tissues that allow these parasites to colonise and persist inside their hosts.

## All roundworms, but with diverse body plans and a plethora of adaptations

Nematoda (roundworms) is a highly diverse phylum of animals with species adapted to an incredible range of environmental habitats and intra-host niches and displaying free-living and parasitic lifestyles [11,12]. The phylum is divided into five (I–V) major phylogenetic Clades [13,14]. Nematodes have conserved gross body plans. The body lacks internal segmentation and is covered with a cuticle that surrounds a body wall composed of an epidermis and a single layer of muscle cells. The mouth is generally terminal and leads to a buccal cavity, which is followed by a pharynx (oesophagus), intestine, and rectum that opens to the exterior by an anus, in females and larvae, and a cloaca, in males. Nematodes have ES and nervous systems, but lack a circulatory system, and most species exhibit sexual dimorphism [15]. The majority of nematodes undergo a similar developmental trajectory, consisting of an egg, four larval stages (L1–L4), and a fifth, adult stage; they all moult four times during development [6]. Many free-living and parasitic nematode species undergo arrested development at the third-stage larvae (L3). These, also called ‘dauer’ larvae, are non-feeding and more resistant to environmental factors than other life cycle stages [6]. They resume development when they are stimulated by suitable environmental triggers such as nutrients and appropriate levels of CO_2_, temperature and pH [6].

Despite their commonalities, the bodies of nematodes exhibit considerable diversity. Tissues and cell types such as body wall muscle, hypodermal and cuticular layers, pharyngeal and ES cells, amphids, phasmids and neurons, and reproductive tracts, are all subject to variation in morphology between and within species throughout their life cycles [15,16]. For example, while the ES system has core and shared functions for the removal of waste products and the release of bioactive molecules, which contribute among other effects to immune evasion and extracorporeal digestion, it differs dramatically in form across species and their developmental stages (Box 1) [16,17]. Moreover, the tissues responsible for excretion–secretion can be functionally redundant, with products originating from either the ES system or other tissues such as the pharynx and anus. For instance, microfilarial stages of key filarial parasites of nonhuman primates display the ES marker phosphatase activity at the mouth, excretory, and anal pores [18]. In contrast, in microfilariae from the human parasite *Brugia malayi*, ES proteins localise at the ES apparatus, which comprises a vesicle that opens to the cuticle via a pore and an excretory cell connecting to the vesicle by a cytoplasmic bridge [19]. The presence/absence and number of tissues, cells and receptors also vary across species. For example, certain parasitic and free-living predatory nematode species have specialised buccal capsules equipped with structures such as teeth, hooks, cutting plates, lancets, or stylets, which facilitate attachment to and feeding from host tissues or prey [15,20,21]. Additionally, the abundance of chemoreceptors in nematodes is correlated with the presence of environmental (extra-host) stages in the life cycle [14,22]. In particular, the model organism *C. elegans*, along with other free-living species, exhibits a high number of chemoreceptors, enabling the sensing of complex external environments. Similarly, parasitic species that navigate external environments for part of their life cycle and with skin-penetrating larvae, such as *Strongyloides* spp. and hookworms (*Ancylostoma* spp. and *Necator americanus*) have a large quantity of these receptors. Meanwhile, single-host, vector-borne, and host-contained parasitic species show significantly reduced chemoreceptor genes [14,22].

Nematode parasitism of vertebrates is thought to be a homoplasious trait with at least five points of origin [12]. As such, parasitic nematodes are not confined to particular nematode lineages and have representatives in Clade I (Dorylaimia), Clade III (Spiruina), Clade IV (Tylenchina), and Clade V (Rhabditina) [12,14], suggesting that physiological adaptations of each species enabled the colonisation of their specific hosts at independent points in the evolutionary timeline.

Despite the significant impact of parasitic nematodes on human and animal health, the majority of our collective knowledge on nematode tissue and cell function and diversity is derived from *C. elegans* [11]. However, among parasitic nematodes are species phylogenetically distant to *C. elegans*, which is reflected in significant diversification of anatomy and tissue function underlain by the expansion of gene families (some of which are absent or not expanded in *C. elegans*) that enabled adaptation to unique biological niches within hosts [14,23]. For instance, the Clade I human (*Trichuris trichiura*) and mouse *(Trichuris muris)* whipworms share only ~50% of gene families with Clade V *C. elegans* [24]. With an evolutionary distance of over 400–500 million years, behaviours and structures and their function in *C. elegans* may have limited translatability to understand the intracellular lifestyle of whipworms inside the intestinal epithelium of their hosts [24,25]. One example of gene families expanded in Clade I parasitic nematodes is the PAN/Apple family with genes potentially involved in host invasion [14]. Even more closely related Clade V species, such as hookworms and the barber’s pole worm (*Haemonchus contortus*), diverged from their common ancestor with *C. elegans* approximately 200 million of years ago [25]. These species have evolved diverse anatomical adaptations that allow hookworms to penetrate the skin [26,27] and both hookworms and pole worms to feed from the blood of their hosts, although using different strategies [20]. In particular, *H. contortus* has a higher copy number of Cathepsin B genes than *C. elegans*, which are potentially involved in its high blood digestion capacity, and has genes encoding acetylcholine-gated receptors (*arc-26* and *arc-27*) and glutamate-sensitive channels (*glc-5* and *glc-6*), which are absent in *C. elegans* and that are possibly anthelminthic targets [28]. Therefore, while *C. elegans* has been instrumental for the understanding of the general physiology of nematodes, the call is now to ‘depart from the model worm’ (Box 2) and adapt and develop parasitic nematode-specific approaches to characterise their functional anatomy and host–parasite interactions [23].

## The unfinished puzzle of parasitic nematode anatomy

Most of our understanding of the anatomy of parasitic nematodes of humans and animals is based on descriptive morphological works performed decades, if not centuries, ago initially to support the taxonomic description of species and their life cycles and aid in diagnostics [29,30]. The majority of these descriptions were generated by using a standard light microscope coupled with a camera lucida to make free-hand drawings [30], some of which are the only whole-organism depiction we have from certain species, and with many now out of print and not easily accessible [29]. Upon the emergence of electron microscopy (EM), a flurry of studies between the 1960s to 1980s provided high-resolution ultrastructural detail of surface (scanning electron microscopy, SEM) and internal (transmission electron microscopy, TEM) adaptations of parasitic nematodes (examples: [31–35]). These studies are, however, often disconnected from the earlier light microscope observations that provide whole-worm context, and are therefore difficult to spatially interpret.

While these morphological studies remain invaluable, with each work having provided a piece of the puzzle of the anatomy of parasitic nematodes, looking back, it is difficult to piece them together into a single coherent image. This is because they represent a patchy menagerie of descriptions with large gaps between species, their different life-cycle stages and the anatomical structures studied, acquired using different techniques and affected by variation in the strains or populations investigated. This is true even for most well-studied and medically and veterinary important species for which we lack complete descriptions of the anatomy of their life cycle stages. Take, for example, human filarial nematodes (*Wuchereria bancrofti, B. malayi* and *Brugia timori*). Morphological changes through parasite development have been described in a smattering of papers from different unknown strains and in a variety of hosts, including cats, dogs, monkeys and humans [36–43]. These descriptions, while pivotal, lack details in areas – for example, in developing larvae which are difficult to retrieve as they are buried in mosquito and mammalian tissues – and are not standardised to enable effective comparisons across studies and species. There is also potential gross variance in growth caused by variation in strains, intermediate mosquito host species, and definitive host species. For *B. malayi* infection of jirds (*Meriones unguiculatus*), the model for human lymphatic filariasis, notes on development were documented in the 1970s [44–46]; however, it was not until 2014 that Mutafchiev *et al*. provided a full description of development following intraperitoneal exposure [47]. While the injection of microfilariae into jirds is not biologically relevant in nature, it has, and continues to be, the main source of *Brugia* laboratory infections, as it produces abundant adult-stage parasites [46]. In another example, most knowledge on the anatomical changes through development of established laboratory models, such as the mouse (*T. muris*) and pig (*Trichuris suis*) whipworms, dates back to their initial life cycle descriptions. These seminal studies provide drawings with stark differences in the level of detail of tissues among similar life stages between species [48–50]. For *T. muris*, there is no proper description of larval stages and images from female and male parasites correspond to L4 larvae instead of adult worms. For *T. suis*, full-body illustrations of L4 and adult stages are missing. Later revisions of *T. muris* growth and moulting by Wakelin [51] and Panesar [52] provide only measurements on total length, without illustrations or pictures, leaving profound gaps in our knowledge of the anatomy of whipworms. Existing gaps in the life cycle stages and their morphology are even more apparent in lesser-studied parasitic nematode species and strains [29].

Why is it important to complete the puzzle of the anatomy of parasitic nematodes? First, revised, detailed and robust anatomical studies will shed light on the spatiotemporal development of key tissues of parasitic nematodes and provide clues on how intra-host niches influence their differentiation and shape. For instance, the stichosome and cuticular inflations of whipworms are not observed in infective L1 larvae, but become apparent in the second larval stage and further develop in the anterior regions of the worm that remain buried within the host intestinal epithelial cells [53], suggesting an influence of mechanical constraints from its intracellular niche on body development. Second, morphological investigations within closely related species with shared anatomy but different life histories will reveal tissue and cell-level differences potentially critical to understanding the role of these structures for the survival of the worms in diverse tissues of their definitive hosts. For example, while very similar morphologically, adult worms of *Trichinella spiralis, Capillaria hepatica*, and *T. trichiura* colonise the small intestine, liver, and large intestine of humans, respectively [54–56]. Thus, it is likely that shared tissues among these species, such as the stichosome, display morphological and functional adaptations that enable them to thrive in such different environments. Ultimately, unravelling tissue function will only be possible with an update of the anatomy of parasitic nematodes for the proper scaffolding and interpretation of next-generation high-resolution imaging and ‘omics’ datasets and the validation of new *in vitro* models of infection.

## Avenues to give function to form

**Figure 1:**
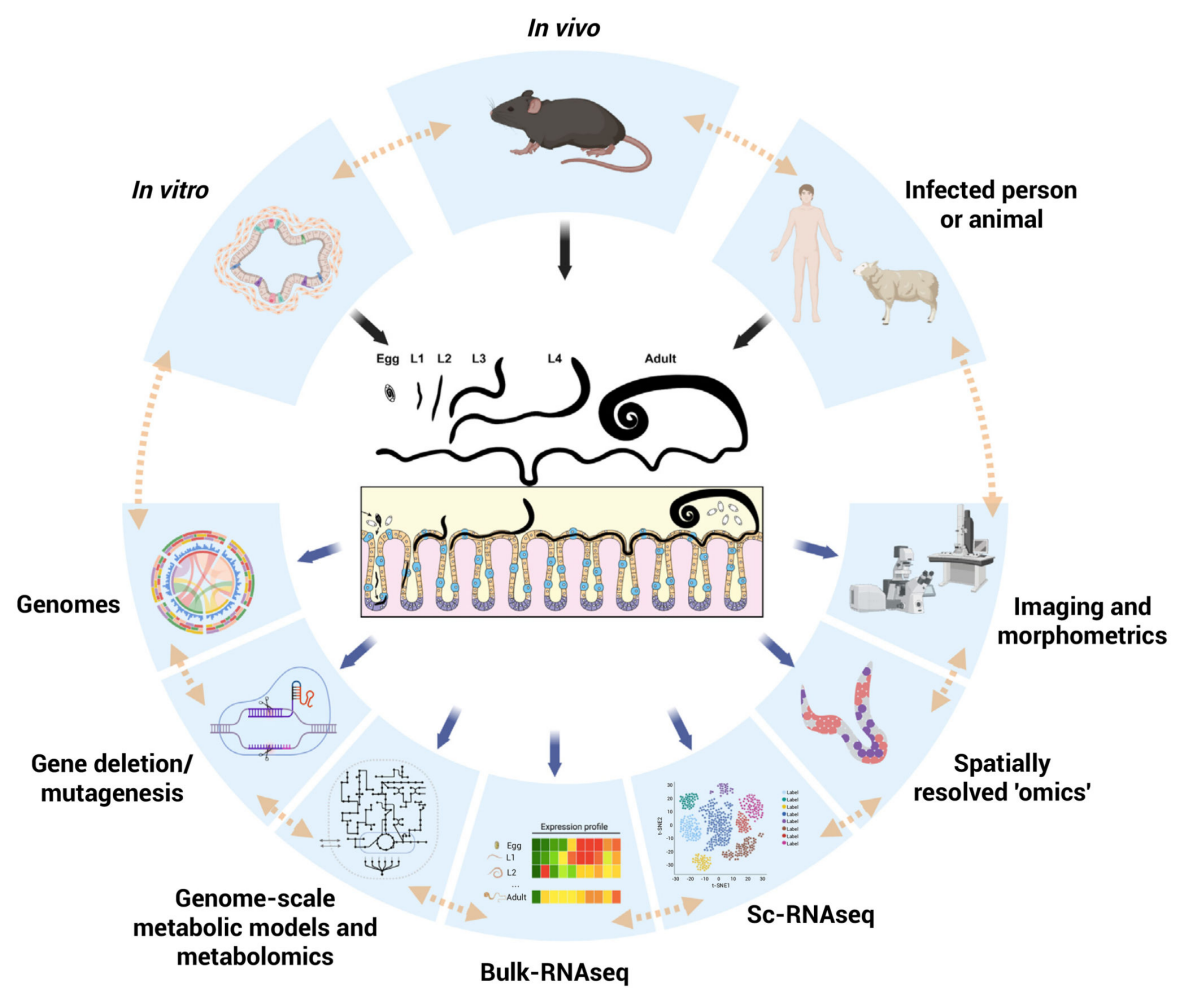
Integrative multidisciplinary approach towards functional anatomy of parasitic nematodes. We propose an interdisciplinary framework through the integration of multiple models and techniques to infer the function of parasitic nematode tissues and cells in space and developmental time. Parasite material (larvae and adults) in isolation or within host tissues is sourced from infected individuals (*ex vivo*, when possible), from livestock and laboratory animals (*in vivo*) or, if existent, from *in vitro* models. The systematic review of the anatomy of life stages of parasites using modern imaging and morphometrics enables the creation of detailed anatomical maps, the characterisation of tissue and cellular morphology, and the visualisation of interactions of parasites with host tissues. These data are crucial for the validation of the development of parasitic nematodes in *in vitro* systems. Moreover, resulting body plans are the backbone for the interpretation of data from spatially resolved ‘omics’, which allocate molecule (RNA, protein, lipid, metabolite) expression profiles of tissues and cellular populations resulting from bulk and single-cell/-nuclei transcriptomic and proteomics studies. Transcriptomic, proteomic and metabolomic data are also incorporated into genome-scale metabolic models initially generated through mining of metabolic reactions in the parasite genomes, resulting in metabolic models at tissue and cellular scales for the different life-cycle stages of the parasites. The identification of essential metabolic pathways for parasite growth and moulting informs the design and improvement of *in vitro* models of parasitic nematodes. Altogether, these approaches result in predictions of the functions of specific tissues of parasitic nematodes. The validation of these predictions is enabled via gene deletion or mutagenesis of parasites and/or the use of inhibitors, blocking antibodies, or the restriction of essential metabolites in *in vivo* and *in vitro* studies, to reveal genes, signalling pathways and biological processes essential to parasite development and survival within their hosts. Figure created using BioRender.

Resolving the morphology of tissues and cells of parasitic nematodes through development is only the first step in the path to ‘give function to form’. We believe this endeavour requires a multidisciplinary approach integrating traditional and modern tools to discover the adaptations and behaviours that enable parasites to thrive and survive within their hosts. In this section, we discuss areas of future research and their complementarity, which we consider key to unlocking the physiology and functional anatomy of parasitic nematodes through space and developmental time (Figure 1).

## *In vivo* and *in vitro* models of infection

Parasitic nematodes have complex life cycles through which different morphological forms of an individual (egg, larvae, adult) are exposed to diverse environmental, vector and/or intermediate and definitive host microenvironments [29]. These morphotypes represent a different phenotypic expression of the same genome [29]. They result from and/or lead to parasite interactions with those microenvironments (outside or inside hosts), which trigger specific signalling pathways and gene expression programmes that enable the development and survival of the individual at each stage. Thus, a deeper understanding of a species’ life cycle, including the anatomy of each developmental stage and the characteristics of the environments it endures, is critical to unravel the origin and function of the tissues that sustain the physiology of parasites.

Obtaining all life-cycle stages of most human and animal parasitic nematodes is, however, difficult, if not impossible. It requires access to intra-host life stages confined to organs that are not easily sampled (e.g., lung, gastrointestinal tract, liver), and has ethical as well as logistical implications, requiring the informed consent of infected individuals and the conservation and transport of material from endemic areas. Thus far, replicating human and animal parasitic nematodes’ life cycles in laboratory animal models has proven challenging for the vast majority of species due to strong host specificity. When feasible, this approach could be problematic as parasites grown in different host species can show intraspecific, host-induced variation in morphology [29,57]. For species with established animal models [58], or for certain veterinary parasites that can be collected from their natural hosts (e.g., [28]), the length and complexity of most parasitic nematode life cycles impose ethical and financial constraints on studies investigating parasite development. Importantly, such research relies on obtaining sufficient biological material at each developmental stage and benefits from work on *in situ* samples. For instance, *T. muris* requires 8 weeks for eggs to embryonate sufficiently to hatch [59], and a further 32 days of infection of mice to reach adulthood and produce an F1 generation [52]. In another example, *B. malayi* requires approximately 14 days to develop from microfilariae to L3 larvae in mosquito hosts, and another 60–71 days to reach sexual maturity in jirds [47]. Nevertheless, controlled infections of sheep with *H. contortus* have enabled transcriptomics studies across its short life cycle (20 days) [28]. Likewise, *Strongyloides* can be cultured in the laboratory by passaging *Strongyloides stercoralis* in dogs (natural host) and jerbils (laboratory model) and *S. ratti* in rats. Unique to these species and those of the *Parastronglyloides* genus is their ability to cycle through a single free-living generation. These features have provided access to all life cycle stages of these species, enhancing our understanding of their anatomy and the gene expression changes (via RNA-seq) across development, and facilitating their genetic manipulation [60–62].

To overcome ethical, logistical, and financial challenges of *ex vivo* and *in vivo* studies, much work is being done to develop *in vitro* culture systems for parasitic nematodes [9]. For example, the epithelial cell line Caco-2 supports invasion, moulting, ecdysis, development to adulthood and reproduction of *T. spiralis* [63]. More recently, co-cultures of ovine and bovine abomasum organoids with L3 larvae of the ruminant gastric parasites *Teladorsagia circumcincta* and *Ostertagia ostertagi* have demonstrated active probing and invasion of the gastric epithelium by the worms. Whilst the larvae did not develop within the organoids, they remained active and survived for several weeks [64,65]. In another model, using murine intestinal organoids, we have effectively recapitulated invasion of the caecal epithelia by L1 *T. muris* larvae. This pioneering system has allowed us to study and visualise the first events of whipworm infection, including mucus degradation, epithelial penetration, and syncytial tunnel formation [66,67]. Excitingly, preliminary data from our laboratory show that *T. muris* can grow and moult up to the L3 stage within this organoid model.

While these results are encouraging, advances in these systems are rather slow. This is in part because of our lack of knowledge on the unique physicochemical conditions (including pH, oxygen levels, metabolite concentrations, mechanical constraints, and tissue architecture) and cellular cues of the host tissues that trigger parasite invasion and development and that determine a successful infection *in vivo*. Thus, the advancement of *in vitro* culture systems for parasitic nematodes requires a better characterisation of their complex host niches, so that key host-restricted features and interactions can be replicated in the laboratory. Once parasite growth and moulting are achieved in a ‘dish’, the resulting life stages require a morphological and molecular validation of species-specific development by comparing them with specimens from the same (when available) or closely related species recovered from infected individuals, livestock or *in vivo* animal models. Since *in vitro* systems are far from fully reproducing the physiological conditions of host organs, developmental differences between parasites grown *in vitro* and those collected *ex vivo* and *in vivo* are expected. Nevertheless, the reductionistic nature of these models can facilitate controlled investigations on the influence of specific host factors on parasite development.

Successful *in vitro* life cycles will be a game changer by expanding the static views on life stages obtained at discrete timepoints from *in vivo* models, with a continuous landscape of *in situ* development in real time. *In vitro* models will enable live imaging and investigation of invasion dynamics, host–parasite interactions, parasite behaviours and the function of critical tissues throughout the infection. Importantly, the broader adoption of these systems will require an extensive characterisation of the models and the standardisation of protocols to support reproducibility across laboratories and enable future comparisons between studies. Together, *ex vivo* samples and *in vivo* and *in vitro* systems are key not only to obtain parasite material for anatomical and omics studies, but also to experimentally validate predictions on signalling and metabolic pathways essential for parasite physiology and metabolism resulting from spatial and metabolic models (see subsequent text).

## Modern imaging technologies and morphometric analysis

To date, most morphological and ultrastructural studies of parasitic nematodes have used a combination of bright-field, fluorescence, as well as SEM and TEM microscopies. These techniques visualise either the surface or internal structure of the worms at different levels of resolution, but not simultaneously; they are restricted to two-dimensional (2D) images, and fail to image whole parasites precluding a holistic map of their body plans [30]. Moreover, while the anatomy of organs and tissues of parasitic nematodes have been roughly described using these tools, the diversity of cellular morphology within these tissues remains understudied.

Modern imaging techniques make it increasingly feasible to create detailed anatomical maps and obtain morphological data at the tissue and cell resolution of whole parasitic nematodes, both in isolation and within their host niches (*in situ*). Confocal and super-resolution microscopy allow acquisition of 3D images of entire worms, capturing external and internal tissues as well as subcellular structures. Examples of the use of these technologies include studies investigating the absorptive function of whipworm tissues [68,69], and more recently, as validation platforms to spatially localise transcripts identified in transcriptomic studies (next section) [23]. Two-photon microscopy enables high-resolution, deep-tissue imaging of internal cellular and subcellular structures and their 3D reconstruction. This technique has lately provided 3D structural detail of the reproductive anatomy of male *Ancylostoma ceylanicum*, including the cloaca, cement gland, spicules and ejaculatory duct [23]. Light-sheet fluorescence microscopy allows much faster acquisition of high-resolution 3D images and reconstruction of thick tissues with organelle detail that can be quantified over time (4D) [30]. This technology has been used to visualise the ES pore pulsing activity and nuclei density of the head of adult *B. malayi* [70]. Fluorescence stereomicroscopy with structured illumination has recently been applied to simultaneously visualise surface topography and internal structures of several parasitic nematodes and produce 3D models [30,71]. X-ray microcomputed tomography can obtain 3D images of a specimen, including its internal structure, by collecting many (typically between 500 and 3000) 2D projections using penetrating X-rays. This technique has been used to capture the spatial positioning of adult *T. muris* within the mouse intestine, revealing the parasite attachment sites to the mucosa [72]. Finally, three-dimensional EM methods – including serial block face, field-emission, and focused ion beam SEM – which allow handling of larger samples, have recently produced ultra-structural maps of entire *T. muris* eggs [73] and larvae within host tissues [66]. Moreover, these technologies have resolved parasite adaptations such as the bacillary cells in adult *T. muris* [69] and head structures and potential ES channels of *B. malayi* [70].

The revision and integration of seminal anatomical studies with new morphological findings arising from state-of-the-art imaging technologies – alongside the inclusion of morphometric measurements for tissues (recent examples: [57,74]) – will yield unprecedented anatomical knowledge of parasitic nematodes. This knowledge will deepen our understanding of tissue ontogeny (morphogenesis, growth and cellular differentiation) across parasitic nematode life cycles as well as species-specific tissue and cellular adaptations that underpin parasitic traits and host-specificity. These detailed morphological descriptions will also be pivotal to draw morphological comparisons between parasites obtained *ex vivo* and *in vivo* and those grown *in vitro*, shedding light on the influence of host tissues and microenvironments on the body plans of parasitic nematodes. Lastly, detailed anatomical maps and morphological data can impact the implementation of and interpretation of data from omics technologies (next section) in two ways. First, information on tissue cellular composition, cell/nuclei size and morphology, and numbers and frequency of cellular populations in different life stages can strongly benefit the design and troubleshooting of single-cell/-nuclei and spatial omics experiments. Specifically, knowledge on the make-up of tissues such as eggshells and worm cuticles can guide the optimisation of protocols for tissue dissociation and sectioning. Further, data on cellular/nuclear size can inform the use of single-cell/-nuclei RNA-sequencing (seq) platforms, as those based on fluidics are limited to specific sizes and can get blocked if the input material is bigger than the range they support, resulting in cell/nuclei loss [75]. Moreover, information on the number of ‘rare’ cells in a tissue of interest can be used to perform statistical power calculations on the total number of cells to be sequenced, and potentially inform decisions for enrichment of tissues before dissociation to ensure the capture of those cells in the experiments. Second, accurate anatomical maps of parasitic nematodes are the foundation for the contextualisation, validation and cross-referencing of omics data. They enable the correct spatial profiling of gene, protein, lipids and metabolite expression for the identification of tissue- and cell-specific molecular signatures. These signatures can reveal previously unrecognised tissues and cell types, as well as the tissues of origin and exchange of ES products and other molecular mediators of host–parasite interactions. Altogether, an improved anatomy of parasitic nematodes will unlock our comprehension of tissue development and function and shed light on processes that enable the successful colonisation of host tissues.

## Spatially resolved ‘omics’ technologies

The advent of genomics and transcriptomics over the last decades has unleashed an unprecedented molecular understanding of parasitic nematodes and enabled comparative genomics of species with related and more distant free-living nematodes [14]. So far, however, the majority of ‘omics’ analyses on parasitic nematodes have focused on bulk studies of pools of whole worms. These studies do not take into account the heterogeneity between individuals, different cell types nor the spatial organisation of these cells [8], making it difficult to ascertain where in the body of the parasite biological processes and pathways play a role.

More recently, advances in the scale of low-input RNA and protein sequencing technologies, allowing tissue and cell resolution maps of gene and protein expression, are opening avenues to functionally characterise tissues of parasitic nematodes. Specifically, bulk RNA or protein sequencing has been performed on tissues or body regions that can be manually dissected or enriched, such as the head, pharynx, intestine and reproductive tissues of the physically large adult *Ascaris suum* [76–78], but also on smaller tissues like the head of *Anisakis* spp. infective larvae [79] and of *B. malayi* females [70]. This targeted approach provides clues on the physiological processes taking place in these tissues but lacks resolution on the cellular heterogeneity and functional diversification of these organs. This resolution is now being achieved with single-cell/-nuclei RNA-seq, resulting in cell-type atlases of gene expression in major tissues of some life cycle stages of a number of parasitic nematode species [23,75,80–83], which are however disconnected/isolated from the body plan organisation. Currently, validation of these findings through morphological visualisation of markers of cellular populations is done via: (i) fluorescent *in situ* hybridisation (FISH) or hybridisation chain reaction (HCR) [23], which is limited to the number of transcripts that can be captured in the tissue and detected using fluorescent microscopy, or (ii) in the best of cases by immunostaining when antibodies targeting parasite proteins are available. Excitingly, new technologies that enable spatial mapping of the transcriptome, lipidome, metabolome or proteome in whole individual worms are becoming available. Specifically, spatial transcriptomics, using cryosection imaging and RNA tomography, laser capture microdissection or 10X Genomics Visium, has been recently applied to female worms of *B. malayi* [70,84]. Moreover, high-resolution atmospheric-pressure matrix-assisted laser desorption/ionization (MALDI) mass spectrometry imaging has been used to visualise anatomical structures and their differential abundances of lipids and saccharides in adults of the flatworm *Schistosoma mansoni* [85] and could be adapted to parasitic nematodes. Further, spatial proteomics via laser capture microdissection and highly sensitive mass spectrometry analysis has been utilised to identify proteins expressed in various tissues of adult females of *Onchocerca volvulus* [86]. As yet, these technologies show low spatial resolution, and spatial proteomics only detects a limited number of proteins compared to spatial transcriptomics. Nevertheless, these fields are rapidly evolving, with spatial technologies that resolve tissue boundaries and quantify molecule expression profiles within tissues at single-cell resolution becoming available. Further, some recent techniques combine spatial transcriptomics and proteomics analyses, enabling integration of unpaired spatial proteomics and single-cell RNA-seq datasets [87]. This higher resolution is critical for locating single cells within tissues of parasitic nematodes, identifying intercellular interactions and allocating biological pathways to specific cellular populations.

The next step to infer the function of tissues and cells in parasite biology will be to apply these tools to spatially resolve host-worm interactions *in situ* across infection; using samples from infected individuals or material derived from *in vivo* and/or *in vitro* laboratory models [88]. Mapping the spatial distribution of both host and parasite cells and their molecules (transcripts, proteins, lipids, metabolites) will reveal inter-species cellular communication and interactions. It will also provide insights into the role of specific parasite tissues at the interface with their host in key infection processes, such as host attachment, invasion, migration, and immune evasion.

## Metabolic models

Across their life cycle, parasitic nematodes need to adapt their metabolism to survive in different host environments, where factors such as nutrients and oxygen could be scarce and conditions hostile due to the immune response mounted by the host [89]. However, we know little about the metabolic pathways underlying these adaptations and even less about the parasite tissues and cells where these critical processes take place.

Genome-scale metabolic models of *C. elegans* have been used to unravel the contribution of nutrients to its metabolism, processes underlying ageing, and the interactions with its microbiota across its development [90–92]. Recently, these models were adapted to *T. muris*, revealing metabolic steps critical for whipworm survival [93], and to *B. malayi*, uncovering metabolic pathways that enable adaptation of the worm to different environments and that underlie its interplay with its bacterial endo-symbiont *Wolbachia* [94]. While initially based on genomic data, these models can incorporate whole-animal bulk RNA-seq transcriptomic data, from worm responses to metabolic gene perturbations [95], life cycle transitions [94] and in the future to toxins and candidates for anthelmintics [96], as well as proteomic and metabolomic datasets if available. Moreover, models could be further refined by integrating single-cell/-nuclei and/or spatial transcriptomic data [97,98], resulting in models at the tissue and cellular scale. These models could predict the metabolic wiring and function of specific parasite tissues, identify enzymes and metabolites that are essential for parasite development and survival inside their hosts, and reveal the metabolic interdependence of host and parasite on their microbiomes (e.g., *Wolbachia* and filarial parasites and whipworms with their host and own microbiota). This knowledge could be exploited for the identification of essential metabolic pathways in parasite growth and moulting, which could inform the improvement of *in vitro* models through the supplementation of critical nutrients and metabolites or alteration of culture conditions (oxygen levels) to resemble those *in vivo*.

## Concluding remarks

We are living in an exciting era in parasitology research where new technologies are providing unprecedented molecular and morphological knowledge of parasitic nematodes, enabling us to rely less on the model worm and focus more specifically on the parasites. The combination of these tools with revised seminal anatomical studies, novel *in vitro* systems of parasite culture, and metabolic models of parasitic nematodes will unlock opportunities for the discovery of the ontogeny and function of tissues throughout parasite development inside their hosts. The concepts and frameworks proposed here are not restricted to parasitic nematodes but are equally applicable to other helminths. With such detailed knowledge, the question remains: how will these novel findings on tissue and cell functional anatomy be integrated for a holistic understanding of the physiology and survival strategies of these parasites within their hosts (see Outstanding questions)?

One important step on the path towards the discovery of traits underlying parasitism is the concerted mining of the vast amount of imaging and omics data resulting from these approaches, which would require the integration and analysis of ‘very big’ datasets through novel computational frameworks. To ensure open access to both the data and the analysis pipelines, the expansion of existing (i.e., WormBase ParaSite, and WormAtlas) and the creation of novel curated central databases will be paramount; an effort funding agencies need to recognise.

In parallel, we parasitologists need to tap into the substantial and detailed knowledge on the physiology and morphology of human and animal organs to understand the microenvironments that parasites encounter during their life cycles. Recently, these fields have enormously benefited from single-cell and spatial omics technologies, resulting in the molecular and metabolic characterisation of human and mouse organs at single-cell resolution. This information is crucial to improve *in vitro* models of parasitic nematodes, but could also be exploited bioinformatically together with the parasite data to predict host-parasite interactions *in situ*.

Importantly, predictions on tissue and cellular function stemming from these studies warrant functional validation. A crucial aspect for mechanistic investigations is the ability to examine the role of specific genes in the physiology and development of parasite tissues. Knockdown of genes with RNA interference (RNAi) has been successfully applied to some parasitic nematodes, but outcomes are often variable in terms of both the extent of gene knockdown and the resulting mutant phenotype [62,99]. Gene deletion or targeted mutagenesis via clustered regularly interspaced short palindromic repeats (CRISPR)/CRISPR-associated protein 9 (Cas9) (CRISPR/Cas9) is the gold standard for functional genomics studies [62,99]. Further, transgenesis enables the generation of fluorescent reporters and biosensors to visualise gene expression patterns, cell morphology and activity, as well as the chemogenetic silencing of specific cells such as neurons [61]. While currently only amenable in a few species, the successful implementation of these genetic tools in *S. stercoralis, S. ratti* and to some extent in *B. malayi* (extensively reviewed elsewhere [61,62,99]), including the generation of stable transgenic lines [100], is paving the way for the adaptation of these technologies to other important parasitic nematode species. This will be transformative for the field, allowing *in vivo* tracking of parasites and shedding light on the adaptations and behaviours crucial for intra-host survival. Alternative approaches, such as inhibitors, blocking antibodies, or restricting essential metabolites in the diet of laboratory animals or in the media of *in vitro* cultures, can also help to ascertain the roles of proteins as well as signalling and metabolic pathways in the function of parasite tissues and in parasite interactions with their hosts. In the future, *in vitro* models will also enable the real-time visualisation of basic physiological processes, such as attachment, feeding and excretion-secretion, using cutting-edge imaging technologies. Furthermore, *in vitro* systems supporting the maintenance and individual tracking of parasites across multiple life stages could facilitate the screening and selection of parasites for genetic modification. This will unlock transgenesis for species without a free-living stage in their life cycle (i.e., *Trichuris* spp.).

A deeper comprehension of the functional anatomy of parasitic nematodes is also setting the stage for future studies on host–parasite coevolution. Comparative ‘omics’ at the tissue and cellular level between closely related species (e.g., among the whipworms *T. muris, T. suis*, and *T. trichiura* or roundworms *A. suum* and *A. lumbricoides*), as recently performed for *C. elegans* and *Caenorhabditis briggsae* [101], will likely uncover key species-specific tissue and cell molecular traits and metabolic programmes critical for the successful infection of their particular hosts. This knowledge, together with detailed anatomical data and a better understanding of the host niches of these parasites, may reveal potential determinants of host-specificity and their evolution.

Ultimately, unravelling species-specific functional anatomy will lead to the development of new avenues of parasite control. The characterisation of the cellular diversity and associated molecular signatures underlying the functions of tissues, such as the neuromuscular, ES and intestinal systems which are targets for anthelmintic therapies, could reveal where specific drug and vaccine targets are located in parasitic nematodes and provide mechanistic explanations for cell sensitivity and resistance to toxic treatments and anthelmintics [82,96]. Moreover, the identification of parasite tissues and cells where immunoregulatory molecules are produced, as well as the anatomical localisation of metabolic processes essential for the survival of parasitic nematodes within hosts, could guide the design of new targeted drugs and vaccines. Similarly, critical drivers and signalling pathways regulating parasite development could be targeted to interrupt early infection or reinfection. Altogether, resolving the function of parasite tissues and cells throughout their life cycle and within their hosts is the key to the discovery of effective interventions to control and treat parasitic nematode infections.

## Box 1 Diversity in the form and functions of nematode excretory–secretory (ES) systems

Nematode excretion–secretion is a critical yet multifaceted function of animal parasitism that acts at the forefront of host–parasite communication [5,17]. Thus, determining the origins of ES products is essential to ascertain the role of different tissues in host–parasite interactions. However, the sources and points of release for ES factors of many parasitic nematodes remain elusive and likely involve more than a single tissue. This is further complicated by the technical difficulty in profiling highly divergent ES structures [16] with few or no similarities to the model organism *C. elegans* (Figure I).

In adult *C. elegans*, four cells (canal, gland, duct, and pore) make up the ES system. The ‘H’ shaped canal cell spans the length of the body that connects posterior to the nerve ring. This channel is linked to a binucleated gland cell, which passes material out of the body through the duct and pore cells (Figure IA) [102]. This system is conserved in the facultative parasite *Pelodera strongyloides* (Figure IB) [16]. Nevertheless, the structure and composition of ES systems in parasitic nematodes is highly diverse and life-stage-dependent to a degree, with larval stages having simpler, more confined and readily apparent excretory structures (Figure IC-F). For instance, ES systems can have large fused canal and gland cells such as in *Ancylostoma caninum* third-stage (L3) larvae (Figure IC) [103] and in *Ascaris lumbricoides* second-stage (L2) larvae [103] and adults (Figure ID) [16]. Further, while the ES apparatus is apparent in some filarial L3 larvae (e.g., *Wuchereria bancrofti*) [39] and a hallmark of microfilariae [43], where it consists of a pore and vesicle leading to a single excretory cell via a cytoplasmic bridge (Figure IE,F), these structures become inconspicuous in later life-cycle stages [36,104]. Instead, in adult stages of *Onchocerca volvulus*, it has been proposed that secretion is mediated through structures within the lateral chord (Figure IG) [105]. Further adaptations are known in Clade I parasitic nematodes (e.g., *Trichuris* spp.), which have no ‘typical’ ES system. Instead the bacillary band is suggested to have potential roles in secretion [32,69] and nutrient absorption [68,106] that likely facilitate growth and persistence of these parasites within the host intestinal epithelia. Spanning below the bacillary band is a single row of large stichocyte cells that make up the stichosome [32,107]. The stichocytes are reported to be connected to bacillary cells through connecting membranes [69,108], and are suggested to accumulate and pass nutrients to the digestive tract [32,68,69]. Moreover, stichocytes show extensive endoplasmic reticulum and have electron-dense ‘secretory’ granules, indicating a role in secretory products generation [32]. As such, the stichocytes are likely both an origin, storage and transit site of immunomodulatory secreted effectors and nutrients [69,109].

With such variety of ES systems across nematodes, the next logical step is to functionally validate the roles of different cells and tissues by locating sites of origin of excreted-secreted products and studying the mechanisms of release over developmental time.

## Box 2 A departure from ‘the model worm’ – the limits of *C. elegans* in parasitological research

The exceptional degree of knowledge on *C. elegans* – enabled by its transparency which has allowed the developmental mapping of every single cell and tissue [110], and an extensive toolkit of genetic and molecular tools that have brought spectacular tractability [111] – makes it a valuable nematode model. However, research on parasitic nematodes that use *C. elegans* suffers from several drawbacks stemming from its lack of traits that facilitate parasitism. Importantly, structural conservation of tissues between *C. elegans* and parasitic nematodes species does not necessarily equate to functional conservation, as these structures may play different roles in supporting species in different environmental and host niches. Even for common and essential organs such as the intestine, recent studies have shown differences in the number, heterogeneity, and functional diversification of intestinal cells between *C. elegans*, closely related clade V *Ancylostoma ceylanicum* and clade III *Ascaris suum* [23,82]. These differences likely reflect adaptations of these parasites for digestion and nutrient acquisition inside their hosts [23,82]. More critically, *C. elegans* cannot be used to study the function of tissues and cells of parasitic nematodes and related parasite-specific gene families, which are absent in free-living worms [14], such as the stichosome and bacillary band present in whipworms and other Clade I nematodes [24]. Similarly, *C. elegans* lacks and therefore cannot model intricate parasite–microbe or parasite–host microbiome interactions, such as *Wolbachia* endosymbionts of filaria [112], or those of gastrointestinal parasitic nematodes and host intestinal microbiomes [113], which are critical for parasite development and long-term survival within hosts. Finally, while *C. elegans* has been an important model for anthelmintic research, it is increasingly recognised that drug-specific outcomes on *C. elegans* versus parasitic nematodes can be drastically different [114]. For instance, macrocyclic lactones (MLs), which affect glutamate-gated chloride channels (GluCLs), lead to pharyngeal pumping inhibition in *C. elegans* and *Haemonchus contortus* at low doses [115,116]. In contrast, the effects of MLs on *Brugia malayi* are linked to channel inhibition in ovaries and surrounding body wall muscle in females and the excretory–secretory pore of microfilaria, likely preventing the release of ES molecules that modulate the host immune response, thereby facilitating host clearance [19,117,118]. Furthermore, the functional annotation of anthelmintic targets using only *C. elegans* can lead to a misunderstanding of the drugs’ mechanisms of action. For example, the mode of action of MLs is thought to be multiplicitous, as are the mechanisms of resistance to these drugs. Mutations in three GluCLs genes (*avr-14, avr-15*, and *glc-1*) cause high levels of resistance in *C. elegans* [119], but those mutations have never been found in a parasitic nematode associated with resistance, and in *H. contortus*, a novel mechanism and gene(s) are clearly driving resistance to ivermectin [120], an important MLs for human and animal parasite control.

Continually using *C. elegans* as a model – because it is anatomically simpler, quicker (3-day life cycle), cheaper, and easy to manipulate in the laboratory [11] – could mislead our understanding of parasitic traits that have evolved to enable the successful survival of parasitic nematodes in their specific environmental or host niches. There is therefore a push to ‘depart from the model worm’ by adapting technological advances from *C. elegans*, such as transgenesis and genetic manipulation [111], and developing parasitic nematode species-specific approaches [23] to shed light on the cellular and molecular diversity and function of tissues that are the basis of parasitism in nematodes.

## Highlights

*Caenorhabditis elegans* is an invaluable model for animal biology, but as a free-living nematode it cannot resolve tissue- and cell-level adaptations unique to parasitic nematodes.

Studies on the structure, function, and development of parasitic nematode tissues are scarce, but could offer clues on how parasitism arose as these organs may be key for host colonisation and intra-host survival.

Revised anatomy of parasitic nematodes using modern imaging is essential to infer tissue function and development.

Detailed parasite morphological data will support the design, troubleshooting, and interpretation of single-cell and spatial omics studies, as well as the validation of *in vitro* parasite cultures.

Functional characterisation of parasite tissues requires *in situ* studies with *in vivo* or *in vitro* models, especially to dissect direct and physical interactions at host–parasite interfaces.

## Outstanding questions

How will novel imaging and omic datasets and metabolic models be mined and integrated for a holistic understanding of parasitic nematode physiology and parasitism?

What human and animal parasitic nematodes are the most important to focus on to understand tissue ontogeny and function throughout life cycles and *in situ* biology? How would we obtain material to perform these studies?

How can key host tissue physicochemical factors, spatial organisation, and cellular cell types needed for parasite development be identified so that they are incorporated in *in vitro* models to support parasitic nematode growth, moulting, and reproduction in a dish?

How will findings on tissue and cell functional anatomy shed light on the evolution of adaptations and behaviours basal to parasitism that enable parasitic nematode development and survival within specific hosts?

## Glossary

Amphids: anterior specialised chemosensory organs of nematodes.
Bacillary band: specialised cuticular region and underlying hypodermis of some Clade I nematode. It contains ~50 000 cuticular pores overlaying hypodermal bacillary cells (approximately 5000 bacillary cells in first and second larval stages).
Camera lucida: an optical device in which rays of light are reflected by a prism to produce an image on a sheet of paper, from which a drawing can be made.
Cuticular inflations: anterior–ventral projections of the cuticle in the bacillary band of whipworms that are thought to be involved in host–parasite interactions.
Embryonation/embryonate: the process of development of the embryo from the starting nucleic acid into first-stage (L1) larvae that occurs inside the nematode egg.
Excretory–secretory (ES) products: soluble mediators released by helminths, either actively exported through secretory pathways or diffused or leaked from the parasite soma as a consequence of physiological processes such as digestion. ES products may interact with host tissues and are involved in parasite invasion and modulation of host immune responses.
Genome-scale metabolic models: mathematical representation of all metabolic reactions within a cell, tissue or organism.
High-resolution atmospheric-pressure matrix-assisted laser desorption/ionization (MALDI) mass spectrometry imaging: a two-dimensional MALDI-mass spectrometric technique used to visualise the spatial distribution of biomolecules, without extraction, purification, separation, or labelling, within sections of biological samples.
Homoplasious: a trait shared by different species but not inherited from their most recent common ancestor. It arises from independent evolutionary origins, often through convergent evolution, rather than shared ancestry.
Laser capture microdissection: a method for isolating specific cells or regions of interest from a tissue sample under direct microscopic visualisation with the help of a laser. This method enables researchers to study these isolated cells or regions in detail, for example, by analysing their DNA, RNA or proteins.
Lateral chord: large epidermal syncytia with multiple closely apposed nuclei that spans the length of the body posterior to the nerve ring.
Microfilaria: pre-first stage (L1) larval forms of filarial nematodes.
Morphotype: distinct form within a parasitic species, distinguished by their physical or structural characteristics. These variations can be related to different life stages, sexes, or even adaptations to different hosts or environments.
Nerve ring: a ring-shaped structure in the central nervous system of nematodes that encircles the oesophagus or pharynx. It is composed of the axonal and dendritic processes of neurons whose cell bodies lie in bundles positioned anterior and posterior to the nerve ring.
Organoid: *in vitro* multicellular clusters resulting from the division of stem cells and containing various differentiated cell types capable of self-renewal and organisation and exhibiting some level of functionality and architecture of the tissue of origin.
Phasmids: posterior chemosensory organs of nematodes.
RNA tomography: a computational method to reconstruct three-dimensional (3D) spatial gene expression patterns from one-dimensional (1D) tomo-seq data. Tomo-seq data are generated by sequencing RNA from cryosections of tissue samples along multiple axes.
Stichosome: an organ present in some Clade I nematodes that encloses the oesophagus and connects with the intestine. It consists of a longitudinal row of glandular cells, the stichocytes, and is suggested to have a role in both nutrient storage and secretion.
